# Coral reef fish predator maintains olfactory acuity in degraded coral habitats

**DOI:** 10.1371/journal.pone.0179300

**Published:** 2017-06-28

**Authors:** Michael Natt, Oona M. Lönnstedt, Mark I. McCormick

**Affiliations:** 1 ARC Centre of Excellence for Coral Reef Studies, James Cook University, Townsville, Queensland, Australia; 2 Department of Marine Biology and Aquaculture, James Cook University, Townsville, Queensland, Australia; 3 Department of Ecology and Genetics, Limnology, Uppsala University, Uppsala, Sweden; Department of Agriculture and Water Resources, AUSTRALIA

## Abstract

Coral reefs around the world are rapidly degrading due to a range of environmental stressors. Habitat degradation modifies the sensory landscape within which predator-prey interactions occur, with implications for olfactory-mediated behaviours. Predator naïve settlement-stage damselfish rely on conspecific damage-released odours (i.e., alarm odours) to inform risk assessments. Yet, species such as the Ambon damselfish, *Pomacentrus amboinensis*, become unable to respond appropriately to these cues when living in dead-degraded coral habitats, leading to increased mortality through loss of vigilance. Reef fish predators also rely on odours from damaged prey to locate, assess prey quality and engage in prey-stealing, but it is unknown whether their responses are also modified by the change to dead-degraded coral habitats. Implications for prey clearly depend on how their predatory counterparts are affected, therefore the present study tested whether olfactory-mediated foraging responses in the dusky dottyback, *Pseudochromis fuscus*, a common predator of *P*. *amboinensis*, were similarly affected by coral degradation. A y-maze was used to measure the ability of *Ps*. *fuscus* to detect and move towards odours, against different background water sources. *Ps*. *fuscus* were exposed to damage-released odours from juvenile *P*. *amboinensis*, or a control cue of seawater, against a background of seawater treated with either healthy or dead-degraded hard coral. Predators exhibited an increased time allocation to the chambers of y-mazes injected with damage-released odours, with comparable levels of response in both healthy and dead-degraded coral treated waters. In control treatments, where damage-released odours were replaced with a control seawater cue, fish showed no increased preference for either chamber of the y-maze. Our results suggest that olfactory-mediated foraging behaviours may persist in *Ps*. *fuscus* within dead-degraded coral habitats. *Ps*. *fuscus* may consequently gain a sensory advantage over *P*. *amboinensis*, potentially altering the outcome of predator-prey interactions.

## Introduction

Although coral reefs are one of the world’s most biologically diverse ecosystems, these habitats are in crisis around the globe, with most showing major signs of degradation [1–3]. The drivers of this ecosystem change are varied and include increased frequency and intensity of severe storms, ocean acidification, thermal bleaching of corals, sedimentation and pollution from land run-off [4–9]. As reefs move from coral-dominated to algal-dominated seascapes, the characteristics of reefs and the resources they provide become modified, leading to relatively rapid changes in the fish community [10, 11]. These changes may in part be due to alterations in the availability of sensory information that fishes rely upon to inform behavioural decisions when the structural complexity and benthic composition of the reef changes [12, 13]. Because many fishes rely on these sources of sensory information, alterations to habitat composition can have repercussions for the efficiency with which they forage and assess risk, ultimately affecting the ability of individuals to survive [12–16].

Many reef fish species rely on olfactory cues to inform risk assessments and learning processes [17]. For example, cues released upon mechanical breakage of the epidermis during a predation event [18–20]. These ‘damage-released’ odours contain numerous chemicals, and combine with currents leading to local and downstream broadcasting of information [17, 21, 22]. To date, responses of fish to damage-released odours has predominantly focussed on prey species such as damselfish (Pomacentridae) [17, 23]. In healthy coral habitats, damselfishes respond innately to the damage-released odours of conspecifics, rapidly switching from foraging to risk-adverse behaviours [12, 17, 23]. The damage-released odour effectively acts as an honest indicator of a predation event in the local environment, and through associative coupling of these cues with predator cues (e.g., visual, chemical) prey learn to identify threats [24]. However, the olfactory responses of some prey species become undermined in dead-degraded coral habitats [12, 14–16, 25]. *P*. *amboinensis* living on dead-degraded coral, overgrown by algae, become unable to detect and respond appropriately to conspecific damage-released odours, unlike those living on healthy coral [14–16, 26]. Indeed, laboratory studies confirm that damage-released odours mixed with small volumes of water that have passed over dead-degraded coral, fail to elicit antipredator responses in *P*. *amboinensis* [27]. Consequently, *P*. *amboinensis* are rendered incapable of developing appropriate risk assessments through direct use of damage-released odour, and do not learn predator identities through associative learning processes [14–16, 28]. *P*. *amboinensis* also stray further away from shelter and have lower activity when living on dead-degraded, compared to healthy, patches of reef [15]. These behavioural changes modify predator-prey dynamics, with the loss of vigilance in prey leading to higher mortality [16, 27–29].

However, the use of damage-released odours is not restricted to prey species, and predators readily exploit the same cocktail of chemicals for their own benefit [30]. The dusky dottyback, *Pseudochromis fuscus* (Pseudochromidae), a small, highly proficient, diurnal predator found throughout the Indo-Pacific [31, 32], forms small territories that often encompass newly-settled *P*. *amboinensis* [31, 33–35]. *Ps*. *fuscus* also exhibits hunting behaviours in response to damage-released odours, released by injured prey such as *P*. *amboinensis*, when wounded by other predators. Damage-released cues from *P*. *amboinensis* also prompt aggregations of *Ps*. *fuscus* in the local environment, promoting prey-stealing activities [30, 36]. As *Ps*. *fuscus* constitute ~10% of the total piscivorous fishes on some reefs [37], and field observations and gut contents analyses show them capable of removing large quantities of prey organisms with great efficiency [31, 38], *Ps*. *fuscus* may significantly impact prey populations within their territories. However, reliance on olfactory cues that may become undecipherable in dead-degraded coral habitats [27, 28], makes *Ps*. *fuscus* an ideal candidate for studying the effects of habitat degradation on a reef fish predator. Since predation is a key process capable of modifying populations, ecosystems and evolution [35, 39, 40], the impact of sensory inhibition on prey fitness and survival is dependent on how predatory counterparts are also affected. If predator success is reduced at a proportional rate to prey escape performance, prey would not be placed at a selective disadvantage to predators. However, if deleterious effects on predators are less than that of their prey counterparts, reduced vigilance could result in population declines in prey species [41].

Few studies have considered the sensory impacts of environmental stressors on coral reef fish predators [41], with most research addressing the effects on prey [15, 27, 28, 42]. Nevertheless, predators are an essential component of predator-prey interactions [17, 43]. The present study aimed to explore whether the presence of a dead-degraded coral habitat affected olfactory-mediated foraging responses of *Ps*. *fuscus* towards damage-released odours of a common prey species, *P*. *amboinensis*, known to be affected by habitat degradation [14–16, 28]. To address this question, the responsiveness of *Ps*. *fuscus* to damage-released odours from *P*. *amboinensis* was tested using a y-maze protocol [41]. Based on previous findings, we predicted that *Ps*. *fuscus* might have problems responding to damage-released odours from *P*. *amboinensis* in water from dead-degraded coral habitat, but not healthy live coral habitat.

## Materials and methods

### Study site and specimen collection

This study was conducted between February and March 2016 at Lizard Island Research Station (LIRS), northern Great Barrier Reef (GBR; 14°40’S, 145°28’E), Australia. *Ps*. *fuscus* (standard length (SL); 58.45 ± 0.61 mm; mean ± SE) from the Lizard Island fringing reef were collected using hand nets and clove oil, from patch reefs 2–6 m in depth. Once caught, fish were transferred to LIRS and relocated into 60 L flow-through aquaria. To prevent aggressive interactions, individuals were separated into 1 L mesh pots within their holding tanks. All individuals were fed daily to satiation with squid and dead juvenile damselfish.

*Ps*. *fuscus* are selective of prey size when hunting [12], likely due to gape limitations [35]. Therefore, settlement size (10.3–15.1 mm SL) *P*. *amboinensis* were selected for the creation of damage-released cues for experiments. These fish were caught at the end of their larval phase using light traps [44], and transferred to a 60 L bin for transport to LIRS, where they were placed in flow-through aquaria with plastic shelters. Fish were fed *Artemia* spp. twice daily to satiation. All procedures were conducted under and approved by James Cook University’s Animal Ethics Committee (approvals A2005, A2080), and all efforts were made to minimize the stress of test organisms. Research at Lizard Island was conducted under approval from the Great Barrier Reef Marine Park Authority (approval G12-35128).

Healthy coral (*Pocillopora damicornis*) and dead-degraded coral covered in a mixture of algae and sessile invertebrates (450–500 ml in volume; dimensions ~200 x 150 x 150 mm) were collected from fringing reefs around Lizard Island, and placed in well-aerated holding tanks of flow-through seawater [15]. Healthy and dead *Po*. *damicornis* are common nursery habitats for newly recruited damselfish, and commonly found within *Ps*. *fuscus* territories [45].

### Experimental protocol

**Fig 1:**
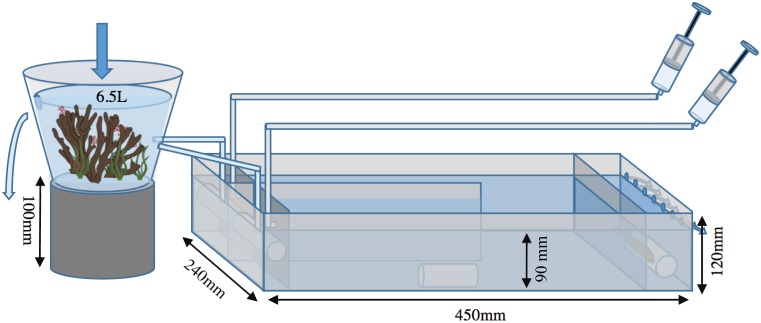
Schematic of the experimental y-maze choice chamber. Diagram shows coral water treatment reservoir, y-maze and cue injection syringes with plumbing.

**Fig 2:**
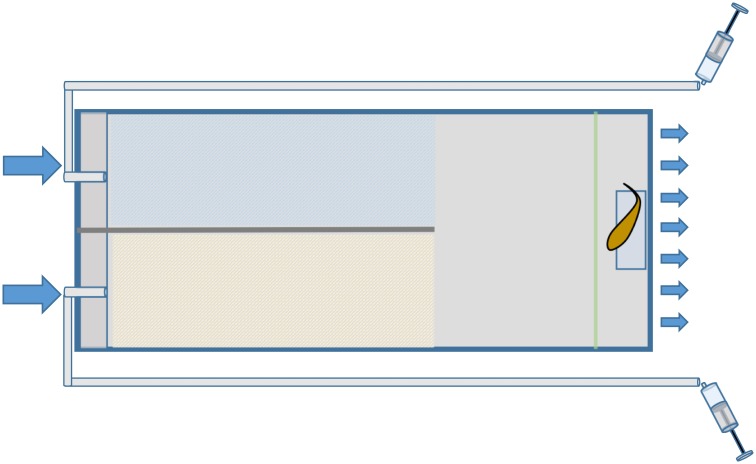
Top-down view of the experimental y-maze choice chamber (450 x 240 x 120 mm). Hatched areas (yellow/blue) indicate the two physically divided chambers (each 270 x 120 x 120 mm). To the right of these is an area (180 x 240 x 120 mm) where the physical barrier (dark grey) is not present, within which a fish acclimation zone (60 x 240 x 120 mm) is created via installation of a rigid removable mesh guard (5 x 5 mm wire mesh) (green). Blue arrows indicate the entrance of water to the y-maze on the left from the water treatment reservoir, and its exit from the y-maze on the right. Syringes and tubes indicate the location of cue injections into chambers.

Olfactory choice trials were conducted in four two-channel choice chambers (y-mazes), similar to those used by Cripps et al. [41] and Lönnstedt et al. [30] (Figs 1 and 2). Seawater pumped from the lagoon was directed into four identical 6.5 L reservoirs. Two reservoirs contained healthy colonies of *Po*. *damicornis*, and two contained dead-degraded coral heads (Fig 1). The volume of material was standardized to 450–500 ml, measured via water displacement, and corals within reservoirs were replaced every three days. Water from reservoirs was gravity fed to y-mazes from a constant height (Fig 1). Water flowed into chambers through a water diffuser, and a rigid mesh (5 x 5 mm gridding) was employed to encourage uniform flow whilst also preventing animal concealment beneath the water diffuser. Water exited the y-maze via several exhaust holes 90 mm up the back wall, allowing water depth to be maintained at 90 mm throughout each chamber (Fig 1). As *Ps*. *fuscus* is often found sheltering on the reef, PVC tubes were placed parallel to the flow in both channels and in the acclimation area. Dye trials were conducted prior to each trial to ensure there was a consistent flow between chambers. Water flow was approximately 25 ml per second, estimated from the flow of water from tubes exiting the reservoir into the y-mazes. The water temperature over trials was 30.4 ± 0.2°C (mean ± SE), measured at the start of each trial.

*Ps*. *fuscus* were not fed 48 h prior to trials to control for satiation, and focal fish were allowed to acclimate in the acclimation zone of y-mazes for a minimum of 4 h, as shown effective by Cripps et al. [41]. At the onset of an eight-minute trial either 15 ml of treatment water mixed with *P*. *amboinensis* skin extracts (a damage-released odour), or treatment seawater alone (a control seawater cue), were injected to one side of the y-maze whilst treatment seawater alone (an injection control) was simultaneously injected into the alternative chamber. This was possible via a 1.2 m long piece of plastic tubing 4 mm in diameter, positioned ~3 cm below the surface of the water, next to the inflow from the reservoir (Figs 1 and 2). Cue injections were completed within 10–15 s and then flushed from injection tubes into chambers via insertion of 60 ml of treatment water to both sides. At the onset of the 60 ml flush, the guard that separated the fish in the acclimation zone from the rest of the choice chamber was slowly removed. Both cue and flush entered the chamber within 30–45 s. Cue and seawater injections occurred twice for each trial: at the start and four minutes into the experiment, as dye trials indicated cues were completely flushed after four minutes. At the end of the trial fish were directed into the acclimation area and the mesh was replaced. The arena was then left to flush for one hour, allowing removal of residual cues from chambers prior to repeating the trial, but with the cue sides reversed. In control trials, treatment seawater without the addition of damage-released odours (i.e., a control seawater cue) was delivered to one side, whilst treatment seawater was again injected into the alternative chamber, to ensure there was no tendency of fish to choose one side or other of the y-maze. All trials were video recorded from above by cameras operated remotely via Wi-Fi, reducing disturbance to animals, and all chambers were screened by a black plastic sheet to minimize visual disturbances. Additionally, as four y-mazes were utilized in the experiment, each treatment was cycled through each maze to spread any positional effects. The side on which the prey-damage cue was initially injected in trials was swapped systematically to account for any possible side bias.

### Skin extract preparation

Newly-settled *P*. *amboinensis* (SL; 12.6 ± 0.01 mm; mean ± SE) were used as donor fish for damage-released odours. Donors were euthanized via cold shock by placing individuals into a seawater ice slurry. Each donor was then placed in a clean petri dish and six similarly sized (~5 mm) superficial cuts were made with a scalpel upon each flank. There was no apparent blood within the extracts and there was no visible difference between damage-released odour extracts and control seawater cues where there had been no addition of damage-released odour. Fish were rinsed in 30 ml of seawater from the relevant coral water treatment and the resulting solution was drawn into a 50 ml syringe ready for injection into the choice chamber (15 ml at the start and 15 ml four minutes into the experiment). Two *P*. *amboinensis* were used for each damage-released cue preparation, as previous studies have shown this to be a sufficient number to elicit successful search behaviours and prey detection, and also accounts for individual variation among donors in terms of cue potency [46]. Donor cues were used within minutes of production in order to minimize the effects of cue degradation [47].

### Video and data analyses

Video-recorded behavioural observations commenced after the mesh guard was removed from the y-maze. To eliminate potential selection by fish for particular chambers, each fish was observed for 16 minutes in total, which combined two eight minute observations because cue side was switched and protocols repeated. Observations were restricted to the two chambers of the y-maze separated by the central partition as dye trials indicated some mixing was apparent beyond this physical division (Figs 1 and 2). The amount of time (in seconds) spent within each chamber within y-maze treatments was recorded. Mean total time spent and visits made to the damage-released odour and seawater cue treatments, for both healthy and dead-degraded coral treatments, was calculated by combining the results from trials both before and after swapping cue sides. The difference in time spent by fish within the cue-injected (either damage-released or seawater control cue) chamber and the alternative seawater injected chamber was calculated by subtracting the time spent in the seawater injected chamber from the time spent in the cue injected chamber. The same calculation was employed to determine the difference in total visitations between chambers within each treatment. The mean difference in time and visitations between chambers in damage-released and seawater control cue treatments was then calculated for each coral water treatment. Trials where the fish did not enter either chamber of the y-maze were removed from analyses as fish were assumed to have failed to acclimate or were not hungry; *n* (healthy water source, damage-released cue) = 1, *n* (healthy water source, control seawater cue) = 4, *n* (degraded water source, damage-released cue) = 3, *n* (degraded water source, control seawater cue) = 1.

### Statistical analyses

Two-way fixed factor analyses of variance (ANOVA) were used to test the effects of water source (water that had either passed over healthy or dead-degraded coral) and cue treatment (damage-released or control seawater cue) on the total time allocation of fish to y-mazes under different coral water treatments. In a similar way, the difference in time allocation to the cue injected (damage-released odour or control seawater cue) and non-cue (seawater) injected chambers of y-mazes under different coral water treatments were also tested. Incorporation of temperature during trials as a covariate had no significant effect on results. Significance was accepted where p < 0.05. Normality and homogeneity of variance were examined using residual analyses and no data transformations were required.

## Results

### Time allocation

**Fig 3:**
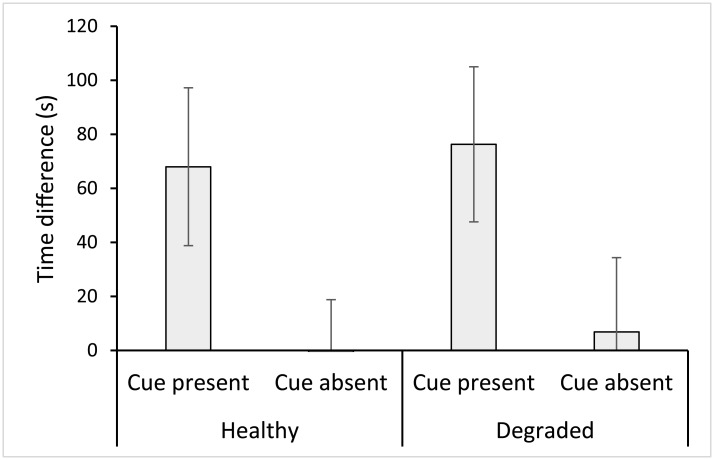
Predator response to prey damage odours, represented as a difference from controls. Mean difference (± SE) in time spent by *Pseudochromis fuscus* between chambers of y-mazes injected with damage-released odours from *Pomacentrus amboinensis* or seawater, when the damage-released odour was present or absent, against a background of either healthy or dead-degraded coral treated seawater; n(left to right) 19, 16, 17, 19.

**Fig 4:**
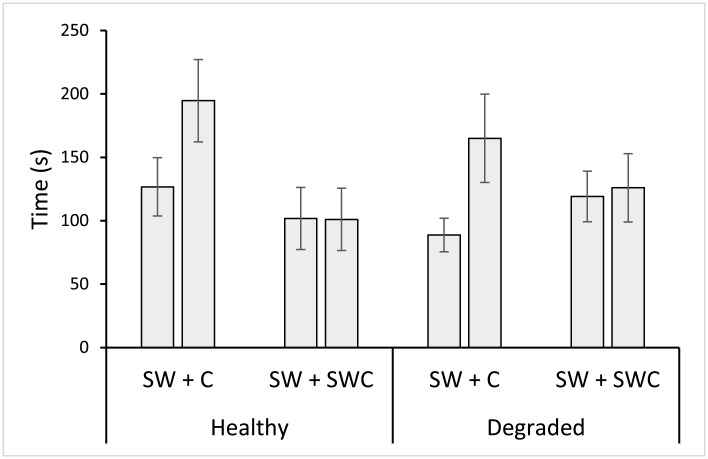
Predator response to prey damage odours or a seawater control. Mean (± SE) time spent by *Pseudochromis fuscus* within chambers of y-mazes injected with seawater (SW) and either damage-released odours from *Pomacentrus amboinensis* (C) or a seawater control cue (SWC), against a background of either healthy or degraded coral treated seawater; n(left to right) 19, 19, 16, 16, 17, 17, 19, 19.

*Ps*. *fuscus* spent a similar total amount of time in the choice chambers of y-mazes, regardless of cue treatment or background water source (Cue F_1, 67_ = 2.054, p = 0.156; Table 1). However, the amount of time fish spent within each chamber of the y-maze varied significantly with cue treatment (Table 2). Analysis of the differences between the time spent in chambers within cue treatments with different background coral treated water sources found a significant effect of cue (F_1, 67_ = 6.630, p = 0.012; Table 2; Fig 3). However, there was no significant effect of water source (Table 2) or interaction between water source and cue (Table 2). Fish spent on average 60 to 80 s more time in the chamber injected with prey damage-released odour compared to the alternative seawater-injected chamber, regardless of water source (healthy or dead-degraded; Figs 3 and 4). However, fish in y-maze chambers where the prey damage-released odour was replaced by a control seawater cue spent an equal amount of time in both sides of the y-maze, in both water sources (Figs 3 and 4). Furthermore, time allocation between chambers injected with seawater control cues was comparable to the seawater injected chamber of y-mazes in trials involving prey damage-released odours (Fig 4).

**Table 1:** Comparison of the total time spent in chambers by *Pseudochromis fuscus* within y-mazes injected with damage-released odours from *Pomacentrus amboinensis* or a control seawater cue, against a background of either healthy or dead-degraded coral treated water. This may be used as a proxy for motivation; *n* (healthy water source, damage-released odour) = 19, *n* (healthy water source, control seawater cue) = 16, *n* (degraded water source, damage-released odour) = 17, *n* (degraded water source, control seawater cue) = 19.

Source	df	Mean Square	F	p
Cue	1	71200.04	2.05	0.16
Water source	1	2848.51	0.08	0.78
Cue * Water source	1	53358.93	1.54	0.22
Error	67	34658.66		

**Table 2:** Comparison of the time differences spent by *Pseudochromis fuscus* to cue injected (prey damage-released odour or control seawater cue) versus seawater injected chambers within y-mazes with either healthy or dead-degraded coral treated background water. *n* (healthy water source, damage-released odour) = 19, *n* (healthy water source, control seawater cue) = 16, *n* (degraded water source, damage-released odour) = 17, *n* (degraded water source, control seawater cue) = 19.

Source	df	Mean Square	F	p
Cue	1	84333.92	6.63	**0.01**
Water source	1	1118.44	0.08	0.77
Cue * Water source	1	2.20	<0.0001	0.99
Error	67	12724.61		

## Discussion

Our findings indicate that dead-degraded coral habitat had no influence on the behavioural responses of *Ps*. *fuscus* to damage-released odours from its prey, *P*. *amboinensis*. Fish in both healthy and dead-degraded coral water treatments increased their time allocation to the chamber of the y-maze injected with damage-released odours, relative to the alternative seawater injected chamber, indicating a clear side preference. However, in control treatments where damage-released odours were replaced by a control seawater cue, fish divided their time equally between chambers, suggesting there was no side preference in the absence of the damage-released odour. These results differed from similar studies conducted on *P*. *amboinensis*, in which anti-predator behaviours in response to conspecific damage-released cues were dampened within dead-degraded coral reef habitats [12]. This suggests that *Ps*. *fuscus* may gain an olfactory advantage over *P*. *amboinensis*, due to the apparent resilience of its olfactory acuity under degraded coral conditions. To establish whether this is the case, future studies should confirm whether *Ps*. *fuscus* actively choose to stalk prey inhabiting degraded over healthy coral environments, where they would benefit from a sensory advantage, and whether capture rates are indeed elevated.

Some evidence already suggests that *Ps*. *fuscus* may preferentially stalk prey in degraded habitats. For example, laboratory-based predation trials between *Ps*. *fuscus* and two species of juvenile damselfish (*Pomacentrus moluccensis* and *Dascyllus aruanus*) in healthy, bleached and degraded algae-covered coral habitats yielded mortality estimates of 25%, 33% and 42%, respectively [29], a trend also supported in the field for *P*. *amboinensis* [34]. Coker et al. [29] suggested that enhanced contrast of prey fish against white bleached corals explains the increased strike rate on prey, but this does not explain the even higher strike rate observed against dark algae-covered corals [29]. Our experiment, and that of Lönnstedt et al. [12, 26], instead suggest that in dead-degraded algae covered coral habitats *Ps*. *fuscus* possess an olfactory advantage over some prey species, perhaps explaining the increased predation rates by *Ps*. *fuscus* observed by Coker et al. [29] and Holmes and McCormick [34]. Nevertheless, although *Ps*. *fuscus* may have a sensory advantage over *P*. *amboinensis*, this is likely not the case for all prey species. Recent research has found that coral-associated species such as *Chromis* sp. and *P*. *moluccensis* are affected similarly to *P*. *amboinensis*, while others, such as *P*. *chrysurus*, *P*. *coelestis*, *P*. *nagasakiensis*, and *P*. *wardi*, maintain their ability to respond appropriately to damage-released odours from conspecifics, even in dead-degraded coral habitats [15, 25, 48]. Therefore, some species may be more vulnerable to predation from *Ps*. *fuscus* within dead-degraded coral habitats than others.

Our results suggest that the reception of information from the damage-released odours remains unmodified in *Ps*. *fuscus*, even when paired with dead-degraded coral treated waters, unlike previous studies on *P*. *amboinensis* [11]. The olfactory failure of *P*. *amboinensis* living in dead-degraded coral habitat has been suggested to be due to interference of the prey’s olfactory receptors by some chemical constituent of water associated with dead-degrading coral [12, 28]. However, our results do not suggest that dead-degraded coral habitats cause antagonism or inhibition of olfactory receptors in *Ps*. *fuscus*, as behaviour did not change between water treatments. An alternative hypothesis suggests that the chemical structures of damage-released cues become modified by small amounts of water from degraded coral habitats [12, 25, 48]. Nevertheless, despite the assumed modification to damage-released odours from *P*. *amboinensis* in our dead-degraded coral water treatments, *Ps*. *fuscus* exhibited foraging behaviours comparable to when within healthy coral water treatments. This result suggests *Ps*. *fuscus* olfactory reception escapes disruption, or fish are capable of utilising odours even when modified by degraded coral waters. Indeed, it may be that damage-released odours that are modified and no longer usable by prey become indicators of profitable opportunities for predators, labelling vulnerable prey within dead-degraded habitat [15]. However, it is also possible that the predator uses some other constituent of the damage-released cue [22], less affected or left unmodified by dead-degraded coral habitat. It is now important to determine the molecular constituents and chemical composition of damage-released odours, to resolve whether foraging in predators and vigilance in prey are informed by the same cues [22, 23]. To establish whether different chemical components are responsible for the differing reactions of predators and prey, future studies should aim to identify these constituents [22].

Previously laboratory experiments have indicated that *Ps*. *fuscus* can evaluate prey quality and size from damage-released odours, informing decision making [30]. Although our results suggest *Ps*. *fuscus* still receives and responds with similar vigour to damage-released cues in dead-degraded and healthy coral habitats, it is unknown whether the quality of information communicated to the predator remains equivalent. It is also unknown whether longer exposures by *Ps*. *fuscus* to dead-degraded coral habitats have a greater impact on their olfactory acuity. In our experiments, *Ps*. *fuscus* were exposed to coral water treatments for a minimum of four hours. However, it may be that effects only become apparent after longer periods of exposure [8, 47, 49, 50]. Since fish may increasingly spend most their life in dead-degraded coral habitats [2], it is important to establish whether chronic exposures differ from short-term. It is also necessary for future research to determine how a range of environmental stressors may influence the sensory ecology of predators [41, 51, 52], and how predator-prey interactions may be affected by multiple simultaneous impacts.

Our findings offer further evidence that predator-prey interactions may become modified by habitat degradation, but are amongst the first to consider the perspective of a coral reef fish predator [29, 41]. Unlike in prey species such as *P*. *amboinensis*, dead-degraded coral habitats appear not to affect the response of *Ps*. *fuscus* to damage-released odours, potentially providing them with an olfactory advantage over their prey. The ramifications of our findings for predator-prey interactions may be significant, especially if confirmed for a range of mesopredators. Future studies should aim to validate the ecological relevance of our findings by confirming whether *Ps*. *fuscus* show an actual preference for hunting *P*. *amboinensis* located on dead-degraded coral habitats, and whether their olfactory advantage leads to increased predation success, or whether prey are able to behaviourally compensate. If prey cannot adapt, increases in predation by species such as *Ps*. *fuscus* may lead to declines in populations of vulnerable reef fish species in certain habitats. Further understanding how environmental stressors influence predator-prey interactions will allow more accurate predictions of the consequences for predator and prey populations, and therefore the reef fish community.

## Supporting information

S1 File Supplementary file- data files. (DOCX)
