# Supplementary material for: Coral reef fish predator maintains olfactory acuity in degraded coral habitats
Source: PLoS One. 2017 Jun 28;12(6):e0179300. doi: 10.1371/journal.pone.0179300 (PMC5489151; doi:10.1371/journal.pone.0179300)
Supplement: S1 File — (DOCX) [file pone.0179300.s001.docx]

Supplementary File- Data files

**Coral Reef Fish Predator Maintains Olfactory Acuity in Degraded Coral Habitats**

Michael Natt, Oona M. Lönnstedt & Mark I. McCormick

| Combined (left and right trial) difference in time/visitation to cue vs. non cue side | | | | |
| --- | --- | --- | --- | --- |
|  |  |  |  |  |
| Treatment | Visitation | | Time | |
| Side | Cue side | Non-cue | Cue side | Non-cue |
| Healthy coral-Damage cue | 33 | 25 | 324 | 145 |
| Healthy coral-Damage cue | 1 | 1 | 1 | 1 |
| Healthy coral-Damage cue | 16 | 24 | 220 | 281 |
| Healthy coral-Damage cue | 44 | 15 | 359 | 132 |
| Healthy coral-Damage cue | 15 | 18 | 85 | 245 |
| Healthy coral-Damage cue | 21 | 25 | 292 | 311 |
| Healthy coral-Damage cue | 11 | 11 | 208 | 203 |
| Healthy coral-Damage cue | 42 | 21 | 294 | 71 |
| Healthy coral-Damage cue | 7 | 3 | 18 | 8 |
| Healthy coral-Damage cue | 21 | 8 | 228 | 38 |
| Healthy coral-Damage cue | 19 | 28 | 100 | 122 |
| Healthy coral-Damage cue | 43 | 25 | 428 | 213 |
| Healthy coral-Damage cue | 42 | 14 | 340 | 147 |
| Healthy coral-Damage cue | 8 | 3 | 28 | 6 |
| Healthy coral-Damage cue | 10 | 2 | 36 | 2 |
| Healthy coral-Damage cue | 6 | 2 | 16 | 10 |
| Healthy coral-Damage cue | 30 | 13 | 401 | 111 |
| Healthy coral-Damage cue | 17 | 10 | 185 | 134 |
| Healthy coral-Damage cue | 10 | 16 | 136 | 227 |
| Healthy coral-Seawater | 4 | 14 | 123 | 255 |
| Healthy coral-Seawater | 10 | 6 | 51 | 29 |
| Healthy coral-Seawater | 5 | 0 | 35 | 0 |
| Healthy coral-Seawater | 3 | 6 | 13 | 70 |
| Healthy coral-Seawater | 19 | 13 | 173 | 147 |
| Healthy coral-Seawater | 1 | 0 | 4 | 0 |
| Healthy coral-Seawater | 11 | 8 | 319 | 181 |
| Healthy coral-Seawater | 1 | 1 | 8 | 1 |
| Healthy coral-Seawater | 12 | 23 | 112 | 246 |
| Healthy coral-Seawater | 6 | 4 | 25 | 12 |
| Healthy coral-Seawater | 25 | 16 | 249 | 109 |
| Healthy coral-Seawater | 8 | 6 | 58 | 85 |
| Healthy coral-Seawater | 1 | 1 | 3 | 6 |
| Healthy coral-Seawater | 3 | 3 | 19 | 24 |
| Healthy coral-Seawater | 20 | 23 | 250 | 318 |
| Healthy coral-Seawater | 6 | 11 | 76 | 158 |
| Healthy coral-Seawater | 10 | 10 | 200 | 90 |
| Dead coral-Damage cue | 7 | 4 | 47 | 31 |
| Dead coral-Damage cue | 8 | 6 | 136 | 80 |
| Dead coral-Damage cue | 20 | 25 | 272 | 157 |
| Dead coral-Damage cue | 4 | 7 | 67 | 58 |
| Dead coral-Damage cue | 19 | 14 | 120 | 122 |
| Dead coral-Damage cue | 6 | 9 | 94 | 153 |
| Dead coral-Damage cue | 10 | 6 | 59 | 112 |
| Dead coral-Damage cue | 52 | 20 | 332 | 100 |
| Dead coral-Damage cue | 7 | 9 | 28 | 45 |
| Dead coral-Damage cue | 39 | 26 | 362 | 159 |
| Dead coral-Damage cue | 6 | 8 | 54 | 29 |
| Dead coral-Damage cue | 10 | 5 | 35 | 19 |
| Dead coral-Damage cue | 39 | 22 | 382 | 113 |
| Dead coral-Damage cue | 40 | 24 | 282 | 158 |
| Dead coral-Damage cue | 19 | 6 | 361 | 71 |
| Dead coral-Damage cue | 2 | 3 | 9 | 12 |
| Dead coral-Seawater | 12 | 14 | 96 | 133 |
| Dead coral-Seawater | 12 | 6 | 451 | 155 |
| Dead coral-Seawater | 16 | 15 | 211 | 312 |
| Dead coral-Seawater | 7 | 11 | 106 | 155 |
| Dead coral-Seawater | 1 | 0 | 1 | 0 |
| Dead coral-Seawater | 12 | 15 | 102 | 141 |
| Dead coral-Seawater | 16 | 21 | 64 | 111 |
| Dead coral-Seawater | 3 | 12 | 23 | 211 |
| Dead coral-Seawater | 13 | 10 | 175 | 134 |
| Dead coral-Seawater | 8 | 4 | 51 | 29 |
| Dead coral-Seawater | 2 | 1 | 7 | 2 |
| Dead coral-Seawater | 10 | 11 | 107 | 233 |
| Dead coral-Seawater | 20 | 18 | 197 | 119 |
| Dead coral-Seawater | 6 | 5 | 41 | 64 |
| Dead coral-Seawater | 3 | 1 | 21 | 9 |
| Dead coral-Seawater | 5 | 9 | 45 | 204 |
| Dead coral-Seawater | 2 | 2 | 209 | 103 |
| Dead coral-Seawater | 9 | 0 | 155 | 0 |
| Dead coral-Seawater | 15 | 11 | 332 | 149 |

| Time difference between chambers (combined L and R trial) | | | Total time in both chambers (combined L and R trial) | |
| --- | --- | --- | --- | --- |
|  |  |  |  |  |
|  |  |  |  |  |
| Treatment | Visitation | Time (seconds) | Visitation | Time (seconds) |
| Healthy coral-Damage cue | 8 | 179 | 58 | 469 |
| Healthy coral-Damage cue | 0 | 0 | 2 | 2 |
| Healthy coral-Damage cue | -8 | -61 | 40 | 501 |
| Healthy coral-Damage cue | 29 | 227 | 59 | 491 |
| Healthy coral-Damage cue | -3 | -160 | 33 | 330 |
| Healthy coral-Damage cue | -4 | -19 | 46 | 603 |
| Healthy coral-Damage cue | 0 | 5 | 22 | 411 |
| Healthy coral-Damage cue | 21 | 223 | 63 | 365 |
| Healthy coral-Damage cue | 4 | 10 | 10 | 26 |
| Healthy coral-Damage cue | 13 | 190 | 29 | 266 |
| Healthy coral-Damage cue | -9 | -22 | 47 | 222 |
| Healthy coral-Damage cue | 18 | 215 | 68 | 641 |
| Healthy coral-Damage cue | 28 | 193 | 56 | 487 |
| Healthy coral-Damage cue | 5 | 22 | 11 | 34 |
| Healthy coral-Damage cue | 8 | 34 | 12 | 38 |
| Healthy coral-Damage cue | 4 | 6 | 8 | 26 |
| Healthy coral-Damage cue | 17 | 290 | 43 | 512 |
| Healthy coral-Damage cue | 7 | 51 | 27 | 319 |
| Healthy coral-Damage cue | -6 | -91 | 26 | 363 |
| Healthy coral-Seawater | -10 | -132 | 18 | 378 |
| Healthy coral-Seawater | 4 | 22 | 16 | 80 |
| Healthy coral-Seawater | 5 | 35 | 5 | 35 |
| Healthy coral-Seawater | -3 | -57 | 9 | 83 |
| Healthy coral-Seawater | 6 | 26 | 32 | 320 |
| Healthy coral-Seawater | 1 | 4 | 1 | 4 |
| Healthy coral-Seawater | 3 | 138 | 19 | 500 |
| Healthy coral-Seawater | 0 | 7 | 2 | 9 |
| Healthy coral-Seawater | -11 | -134 | 35 | 358 |
| Healthy coral-Seawater | 2 | 13 | 10 | 37 |
| Healthy coral-Seawater | 9 | 140 | 41 | 358 |
| Healthy coral-Seawater | 2 | -27 | 14 | 143 |
| Healthy coral-Seawater | 0 | -3 | 2 | 9 |
| Healthy coral-Seawater | 0 | -5 | 6 | 43 |
| Healthy coral-Seawater | -3 | -68 | 43 | 568 |
| Healthy coral-Seawater | -5 | -82 | 17 | 234 |
| Healthy coral-Seawater | 0 | 110 | 20 | 290 |
| Dead coral-Damage cue | 3 | 16 | 11 | 78 |
| Dead coral-Damage cue | 2 | 56 | 14 | 216 |
| Dead coral-Damage cue | -5 | 115 | 45 | 429 |
| Dead coral-Damage cue | -3 | 9 | 11 | 125 |
| Dead coral-Damage cue | 5 | -2 | 33 | 242 |
| Dead coral-Damage cue | -3 | -59 | 15 | 247 |
| Dead coral-Damage cue | 4 | -53 | 16 | 171 |
| Dead coral-Damage cue | 32 | 232 | 72 | 432 |
| Dead coral-Damage cue | -2 | -17 | 16 | 73 |
| Dead coral-Damage cue | 13 | 203 | 65 | 521 |
| Dead coral-Damage cue | -2 | 25 | 14 | 83 |
| Dead coral-Damage cue | 5 | 16 | 15 | 54 |
| Dead coral-Damage cue | 17 | 269 | 61 | 495 |
| Dead coral-Damage cue | 16 | 124 | 64 | 440 |
| Dead coral-Damage cue | 13 | 290 | 25 | 432 |
| Dead coral-Damage cue | -1 | -3 | 5 | 21 |
| Dead coral-Seawater | -2 | -37 | 26 | 229 |
| Dead coral-Seawater | 6 | 296 | 18 | 606 |
| Dead coral-Seawater | 1 | -101 | 31 | 523 |
| Dead coral-Seawater | -4 | -49 | 18 | 261 |
| Dead coral-Seawater | 1 | 1 | 1 | 1 |
| Dead coral-Seawater | -3 | -39 | 27 | 243 |
| Dead coral-Seawater | -5 | -47 | 37 | 175 |
| Dead coral-Seawater | -9 | -188 | 15 | 234 |
| Dead coral-Seawater | 3 | 41 | 23 | 309 |
| Dead coral-Seawater | 4 | 22 | 12 | 80 |
| Dead coral-Seawater | 1 | 5 | 3 | 9 |
| Dead coral-Seawater | -1 | -126 | 21 | 340 |
| Dead coral-Seawater | 2 | 78 | 38 | 316 |
| Dead coral-Seawater | 1 | -23 | 11 | 105 |
| Dead coral-Seawater | 2 | 12 | 4 | 30 |
| Dead coral-Seawater | -4 | -159 | 14 | 249 |
| Dead coral-Seawater | 0 | 106 | 4 | 312 |
| Dead coral-Seawater | 9 | 155 | 9 | 155 |
| Dead coral-Seawater | 4 | 183 | 26 | 481 |
